# Concentration of hospital utilization in the capital area among elderly inpatients from non-capital regions in South Korea: Before and during the COVID-19 pandemic

**DOI:** 10.1371/journal.pone.0336744

**Published:** 2026-08-14

**Authors:** Yeaeun Kim, Jihoon Lee, Jongho Park

**Affiliations:** 1 Department of Health Care Management, Catholic University of Pusan, Busan, South Korea; 2 Department of Medical Management, Pusan National University, Busan, South Korea; 3 Department of Health Care Management, Daegu University, Gyeongbuk, South Korea; Sungkyunkwan University - Suwon Campus: Sungkyunkwan University - Natural Sciences Campus, KOREA, REPUBLIC OF

## Abstract

Hospital utilization in South Korea exhibits a significant concentration in the capital area, posing a persistent social issue. Given the heightened vulnerability of elderly patients to COVID-19, their continued travel to capital-area hospitals during the pandemic could have facilitated rapid virus dissemination, necessitating a review of the regional healthcare delivery system. This study therefore aimed to analyze the sustained concentration of hospital utilization in the capital area among regional elderly patients and to identify the characteristics of those who utilized these facilities during the pandemic. Data for this study were obtained from the Korea National Hospital Discharge In-Depth Injury Survey (KNHDIS) for the periods 2018–2019 (pre-COVID-19) and 2020–2021 (during COVID-19). A total of 6,026,155 weighted patient data points for non-capital area residents aged over 65 years were analyzed. Statistical analyses, including chi-square tests and logistic regression, were conducted using SAS 9.4 to examine changes in capital-area hospitalization and to identify the characteristics of the study subjects during the pandemic. The overall rate of capital-area hospitalization among regional elderly patients remained stable at 7.5% both before and during the COVID-19 pandemic. However, detailed results revealed statistically significant shifts across specific age groups and main diagnoses, with increases observed in some characteristics. Logistic regression analysis indicated that during the pandemic, regional elderly patients with diagnoses of cancer, higher disease severity (CCI > 3 points), and those undergoing surgery demonstrated a significantly higher likelihood of hospitalizing in the capital area compared to their respective comparison groups. The observed persistent concentration of hospitalizations in the capital area necessitates the urgent implementation of governmental policies aimed at revitalizing regional medical systems. Furthermore, hospitals located in non-capital areas are urged to strategically invest in facilities and advanced medical devices and develop targeted strategies to attract regional patients, considering the identified characteristics of elderly regional patients who utilize capital-area hospitals.

## Introduction

Equitable access to quality healthcare remains a pressing global challenge despite advancements in medical science [1]. Across both developed and developing countries, healthcare resources—such as facilities, professionals, and technologies—tend to concentrate in urban centers, leaving rural and underserved populations with limited access [2,3]. This uneven distribution contributes to persistent health disparities shaped by geography, socioeconomic status, and other social determinants. Global research increasingly highlights how structural inequality and spatial patterns intersect, calling for targeted policies that ensure fair and inclusive healthcare access for all. [4]

Despite its remarkable economic growth and social development, South Korea is not exempt from these global challenges of healthcare inequity. The country operates a three-tier healthcare delivery system consisting of primary, secondary, and tertiary levels of care. Primary care is mainly provided by local clinics and public health centers, while secondary care is delivered by small to medium-sized hospitals that handle general inpatient and specialist services. Tertiary care, however, is concentrated in large general hospitals located predominantly in the Seoul Metropolitan Area (SMA), equipped with advanced facilities and subspecialty departments. As a result, non-capital regions are primarily served by primary and secondary institutions with limited healthcare capacity, which often lack the resources and expertise to manage the complex and severe conditions common among older adults. In addition, regional shortages of healthcare professionals and restricted transportation options collectively constrain access to local medical services. Consequently, many elderly individuals in non-capital regions are compelled to seek care in the capital area, where advanced facilities and specialists are concentrated. This reflects a systemic imbalance in the healthcare delivery structure and deepens long-standing regional disparities in access to care. While the country boasts a universal health insurance system and has made considerable strides in improving overall health outcomes, a significant and persistent issue is the hyper-concentration of healthcare resources in its capital area, particularly in the Seoul Metropolitan Area (SMA) [5,6]. Critically, advanced hospitals, specialized medical professionals, and cutting-edge technologies are overwhelmingly situated in the SMA. This metropolitan centralization not only draws patients away from non-metropolitan regions, but also raises patient burdens and undermines the sustainability of regional healthcare institutions [7,8]. The geographic concentration of advanced hospital facilities in the SMA, therefore, represents not merely a distributional characteristic but a structural outcome shaped by decades of demographic, economic, and policy-driven development—one that continues to drive healthcare-seeking behavior among elderly patients residing in non-capital regions.

During the COVID-19 pandemic, the South Korean government implemented strict social distancing measures and actively discouraged inter-regional movement to contain the virus’s spread [9]. These restrictions were particularly critical for older adults, who faced significantly higher risks of severe illness and mortality from COVID-19 [10,11]. For this vulnerable population, minimizing travel and utilizing healthcare services within their residential area was strongly encouraged to reduce potential exposure. However, if elderly patients in non-capital regions persisted in concentrating their hospital visits in the capital area during the pandemic, this behavior could have not only undermined infection control efforts but also highlighted persistent structural weaknesses in the regional healthcare delivery system. Therefore, it is essential to investigate whether such patterns of capital-area utilization persisted during COVID-19, and how they compare to pre-pandemic trends, to evaluate the effectiveness of existing healthcare policies and regional response strategies.

Previous studies on elderly patients’ hospital utilization during the COVID-19 pandemic have primarily focused on changes in service use and delays in treatment based on patient characteristics. For example, Park et al. [12] reported that elderly patients with chronic diseases maintained more consistent hospital visits compared to those with respiratory conditions. Farzad et al. identified demographic and socioeconomic disparities in hospital utilization, highlighting the influence of sex, age, and insurance status [13]. Other studies have similarly examined how individual-level factors shaped care-seeking behavior during the pandemic [14–18]. However, few studies have explored how the inter-regional movement of elderly patients—specifically between capital and non-capital areas—changed during COVID-19, despite its implications for infection control and healthcare equity.

Accordingly, this study aims to examine the extent of inpatient healthcare utilization concentrated in the capital area by analyzing and comparing hospitalization patterns among individuals aged 65 and older residing in non-capital regions before and during the COVID-19 pandemic. In particular, it seeks to determine whether the pattern of capital-area hospital use among non-capital elderly patients persisted during the pandemic, despite government measures to restrict regional movement. Additionally, the study explores the demographic and clinical characteristics associated with capital-area hospitalization among this population. By identifying the extent and nature of such concentration, the study intends to provide empirical evidence to support policy efforts directed at mitigating regional disparities in healthcare access and enhancing the resilience of the healthcare delivery system under public health emergencies.

## Materials and methods

### Data source and preparation

In this study, we used data from the Korea National Hospital Discharge In-Depth Injury Survey KNHDIS), provided by Korea Disease Control and Prevention Agency (KDCA). Ethical approval was not required for this study as it exclusively utilized publicly available, de-identified dataset provided by the government agency that does not involve any interaction with human subjects.

The dataset comprised 1,191,791 patient discharges from general hospitals nationwide, covering pre-COVID-19 (2018–2019) and during-COVID-19 (2020–2021) periods. From the initial dataset, we extracted 423,551 raw data points for hospitalized patients aged 65 years or older. Subsequently, 261,833 records were selected, limited to patients residing in non-capital areas, excluding the Seoul Metropolitan Area (SMA). Finally, to analyze broader healthcare utilization patterns separate from primary COVID-19 treatment, 261,095 data points were retained after excluding cases with a main diagnosis of COVID-19 NOS (U07.1).

**Fig 1:**
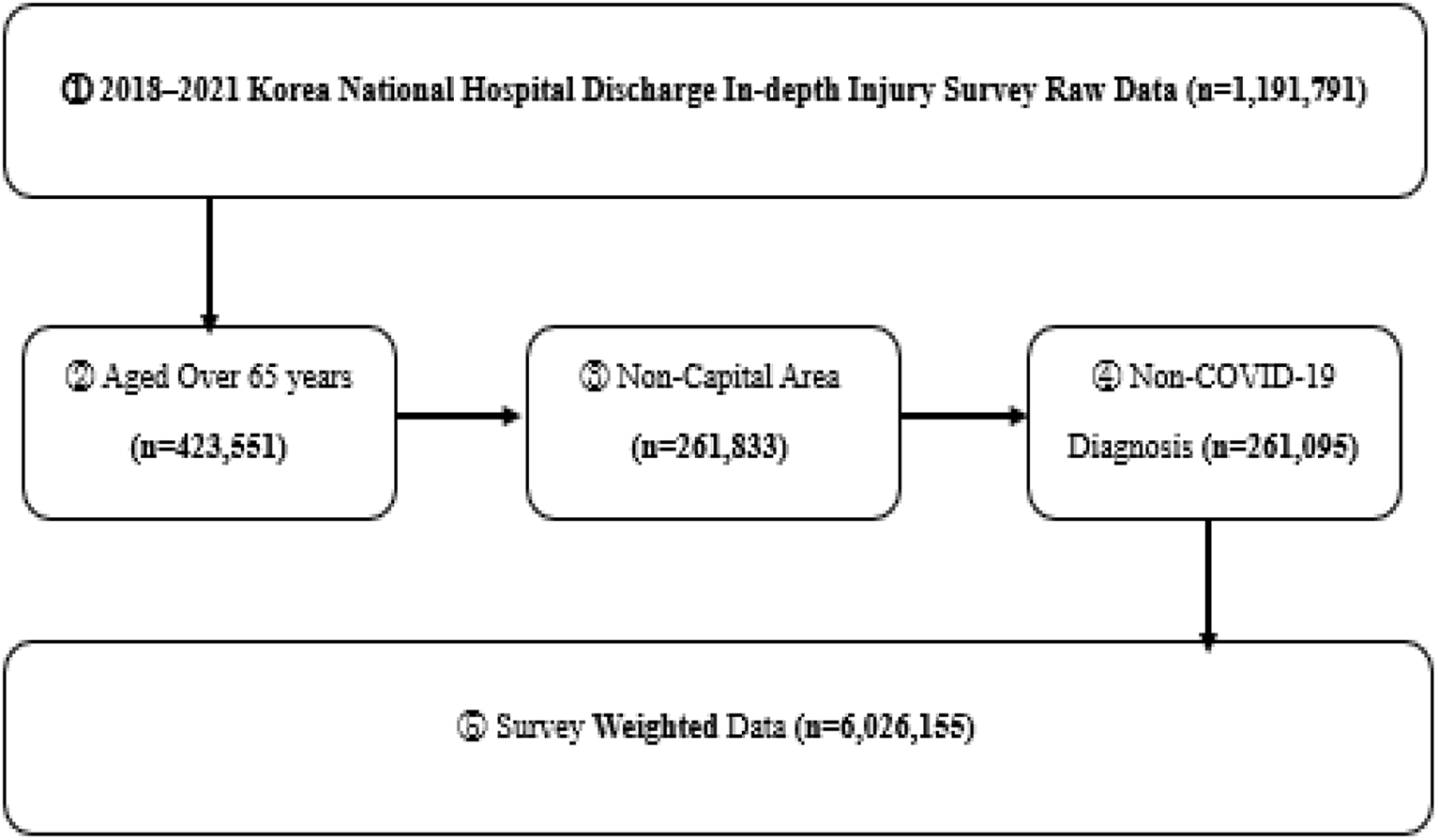
Flow chart to select the number of study samples.

The KNHDIS employs a complex sample design, necessitating the application of sampling weights for accurate population estimates. Specifically, the survey uses a two-stage stratified cluster sampling design, in which general hospitals are selected as primary sampling units (PSUs) after stratification by hospital bed size and geographic region, and discharged patients within sampled hospitals are subsequently selected as secondary sampling units (SSUs) using systematic sampling [19]. Therefore, we applied KDCA-provided weights to the selected data, resulting in an estimated 6,026,155 weighted cases for analysis [20–22]. Fig 1 illustrates the detailed data selection workflow.

### Variables

The dependent variable was Hospitalization Location in the Capital Area (HLCA) for inpatients aged 65 years or older residing in non-capital regions. This binary variable was coded as “1” if the patient’s hospitalization occurred in the Capital Area and “0” if hospitalization occurred within their non-capital residential area.

Independent variables included the hospitalization period (pre- or during-COVID-19), patient demographics (sex, age, and residential area), and clinical characteristics such as main diagnosis, surgical status, and severity of illness as measured by the Charlson Comorbidity Index. These variables were selected to identify factors associated with capital-area hospitalization among elderly patients from non-capital regions.

### Data analysis

We employed both descriptive and inferential statistical methods to analyze inpatient healthcare utilization patterns. A chi-square test was performed to assess differences in capital-area hospitalization among elderly patients from non-capital regions, comparing pre-pandemic (2018–2019) and during-pandemic (2020–2021) periods. To identify characteristics associated with capital-area hospitalization during COVID-19, we utilized a logistic regression model. Statistical significance for all analyses was determined using a two-tailed test with a p-value of < 0.05, and results are presented with 95% confidence intervals. All data collection, cleaning, and statistical analyses were conducted using SAS 9.4 (SAS Institute, Inc., Cary, NC, USA). All analyses incorporated the complex survey design of the Korea National Hospital Discharge In-depth Injury Survey and were performed using the official sampling weight variables provided by KDCA to account for stratification, clustering, and unequal selection probabilities.

## Results

### Characteristics of Study Population

Table 1 presents the distribution of hospitalization locations for elderly patients (aged 65 years or older) residing in non-capital areas of South Korea, comparing the periods before (2018–2019) and during (2020–2021) the COVID-19 pandemic. The data, derived from weighted cases, illustrates the proportion of hospitalizations occurring in the capital area versus those within the patients’ residential (non-capital) areas. The percentage of hospitalizations in the capital area remained consistent at 7.5% in both the pre-pandemic and during-pandemic periods, indicating no statistically significant change in capital-area utilization among this population (P = 0.21).

**Table 1:** Distribution of Hospitalization Location for Elderly Patients Residing in Non-Capital Areas, Before and During the COVID-19 Pandemic (Weighted Data).

Variable	Total of hospitalization		Hospitalization in the capital area		Hospitalization in the residential areas		p-value
	N	%	N	%	N	%	
Before COVID-19 (2018–2019)	3,145,064	52.2	235,156	7.5	2,909,908	92.5	0.21
During COVID-19 (2020–2021)	2,881,091	47.8	214,643	7.5	2,666,448	92.6	

### Characteristics Associated with Hospitalization Location in the Capital Area

Table 2 presents the detailed distribution of Hospitalization Location in the Capital Area (HLCA) rates among elderly patients (aged 65 years or older) residing in non-capital regions, stratified by gender, age, and specific residential area, both before (2018–2019) and during (2020–2021) the COVID-19 pandemic.

**Table 2:** Capital Area Hospitalization Rates by Patient Characteristics Before and During the COVID-19 Pandemic (Weighted Data).

Variable	Total of hospitalization*	Hospitalization Before COVID-19 (2018–2019)			Hospitalization During COVID-19 (2020–2021)			p-value
		Capital area		Residential area	Capital area		Residential area	
	N	N	%	N	N	%	N	
**Gender**								
Male	2,842,252	119,301	8.1	1,345,577	110,613	8.0	1,266,760	<.01
Female	3,183,902	115,854	6.9	1,564,331	104,029	6.9	1,399,688	0.42
**Age (years)**								
65–74	2,831,694	128,175	8.9	1,312,547	124,843	9.0	1,266,130	0.02
75–84	2,420,414	87,962	6.7	1,218,968	74,185	6.7	1,039,299	0.04
Over 85	774,046	19,019	4.8	378,394	15,615	4.2	361,019	<.01
**Residential Area**								
Chungbuk	274,980	22,270	14.7	129,177	22,603	18.3	100,929	<.01
Chungnam	731,144	54,971	14.4	326,824	50,408	14.4	298,940	0.70
Gangwon	405,658	27,008	12.3	192,972	26,167	14.1	159,511	<.01
Jeonbuk	526,847	22,850	8.2	255,841	19,059	7.7	229,096	<.01
Jeonnam	915,091	34,095	7.2	438,237	29,487	6.7	413,272	<.01
Gyeongbuk	1,117,935	36,137	6.1	553,481	31,967	6.1	496,351	0.09
Jeju	168,968	5,573	6.0	87,509	5,212	6.9	70,674	<.01
Gyeongnam	1,885,531	32,252	3.4	925,867	29,738	3.2	897,674	<.01
**Main Diagnosis**								
Certain infectious and parasitic diseases (A00-B99)	268,274	4,413	3.0	142,116	3,020	2.5	118,725	<.01
Neoplasms (C00-D48)	768,824	59,090	15.4	324,190	61,403	15.9	324,141	<.01
Diseases of the blood and blood-forming organs and certain disorders involving the immune mechanism (D50-D89)	36,017	1,178	6.2	17,855	869	5.1	16,115	<.01
Endocrine, nutritional, and metabolic diseases (E00-E90)	173,573	3,923	4.3	87,290	2,561	3.1	79,799	<.01
Mental and behavioral disorders (F00-F99)	21,185	948	7.5	11,682	526	6.2	8,028	<.01
Diseases of the nervous system (G00-G99)	117,069	6,289	9.7	58,833	5,322	10.3	46,626	<.01
Diseases of the eye and adnexa (H00-H59)	122,065	6,946	11.0	56,089	5,066	8.6	53,964	<.01
Diseases of the ear and mastoid process (H60-H95)	70,024	2,330	6.4	34,024	1,463	4.4	32,207	<.01
Diseases of the circulatory system (I00-I99)	782,580	28,523	7.1	374,314	26,649	7.0	353,094	0.28
Diseases of the respiratory system (J00-J99)	576,978	13,271	3.7	349,986	9,325	4.4	204,395	<.01
Diseases of the digestive system (K00-K93)	602,189	16,914	5.6	285,614	15,157	5.1	284,504	<.01
Diseases of the skin and subcutaneous tissue (L00-L99)	48,210	1,398	5.4	24,626	900	4.1	21,286	<.01
Diseases of the musculoskeletal system and connective tissue (M00-M99)	507,580	22,899	9.0	232,730	21,037	8.4	230,914	<.01
Diseases of the genitourinary system (N00-N99)	384,719	9,343	4.7	191,025	7,465	4.1	176,887	<.01
Congenital malformations, deformations, and chromosomal abnormalities (Q00-Q99)	3,594	361	21.0	1,359	290	15.5	1,584	<.01
Symptoms, signs, and abnormal clinical and laboratory findings, NEC (R00-R99)	189,674	4,428	4.3	98,437	2,718	3.1	84,090	<.01
Injury, poisoning, and certain other consequences of external causes (S00-T98)	823,304	16,932	4.0	404,746	12,900	3.2	388,725	<.01
Factors influencing health status and contact with health services (Z00-Z99)	530,296	35,970	14.3	214,992	37,970	13.6	241,364	<.01
**Surgery**								
NOT Performed	4,596,342	161,346	6.6	2,282,097	143,174	6.7	2,009,725	0.04
Performed	1,429,812	73,809	10.5	627,811	71,469	9.8	656,723	<.01
**CCI**								
0 point	4,411,104	145,965	6.4	2,149,413	131,849	6.2	1,983,877	<.01
1–2 points	1,129,093	47,149	7.8	555,758	44,267	8.4	481,919	<.01
Over 3 points	485,957	42,041	17.0	204,736	38,527	16.1	200,652	<.01
* Weighted patient counts may not exactly match the total of 6,026,155 due to rounding after applying sampling weights								

Statistically significant changes were observed in HLCA rates based on several factors. Males experienced a slight decrease in capital-area hospitalization (8.1% to 8.0%, p < 0.01). By age group, patients aged 85 years or older demonstrated a significant decrease (4.8% to 4.2%, p < 0.01).

Regarding residential area, Chungbuk province showed a significant increase in HLCA from 14.7% to 18.3% (p < 0.01), maintaining the highest proportion. Conversely, HLCA rates significantly decreased for residents of Jeonbuk, Jeonnam, and Gyeongnam provinces (p < 0.01).

During the COVID-19 pandemic, certain diagnostic categories exhibited increased hospitalization in the capital area, notably for neoplasms (C00–D48) which rose from 15.4% to 15.9%, diseases of the nervous system (G00–G99) increasing from 9.7% to 10.3%, and diseases of the respiratory system (J00–J99) rising from 3.7% to 4.4%. In contrast, HLCA rates decreased across the remaining 15 diagnostic categories during the pandemic, with the most pronounced decline observed in congenital malformations, deformations, and chromosomal abnormalities (Q00–Q99), dropping significantly from 21.0% to 15.5%. For cases where surgery was performed, the HLCA rate significantly decreased from 10.5% to 9.8%, while for the Charlson Comorbidity Index (CCI), HLCA decreased in the 0-point (6.4% to 6.2%) and >3-point groups (17.0% to 16.1%) but significantly increased for the 1–2-point group (7.8% to 8.4%) (all p < 0.01).

### Factors associated Capital Area Hospitalization during the COVID-19

Table 3 presents the results of a logistic regression analysis identifying factors associated with the odds of hospitalization in the capital area among elderly patients residing in non-capital regions during the COVID-19 pandemic. Females had significantly higher odds (OR = 1.044) of being hospitalized in the capital area compared to males (reference group). Younger elderly patients showed significantly higher odds of capital area hospitalization compared to those aged over 85 years (reference group). Specifically, patients aged 75–84 years had 1.456 times higher odds, and those aged 65–74 years had 1.900 times higher odds.

**Table 3:** Odds Ratio (ORs) and 95% Confidence Intervals (CIs) for Capital Area Hospitalization During the COVID-19 Pandemic (2020–2021).

Variable	Hospital utilization in the capital area during COVID-19 (2020–2021)			
	Odds ratio	95% CI		p-value
		Low	High	
**Gender**				
Male	1			
Female	1.044	1.035	1.054	<.01
**Age (years)**				
Over 85	1			
75–84	1.456	1.429	1.482	<.01
65–74	1.900	1.867	1.935	<.01
**Residential Area**				
Gyeongnam	1			
Gyeongbuk	2.168	2.133	2.204	<.01
Jeonnam	2.563	2.520	2.606	<.01
Jeju	2.659	2.577	2.743	<.01
Jeonbuk	2.995	2.938	3.053	<.01
Gangwon	5.389	5.294	5.487	<.01
Chungnam	5.447	5.364	5.530	<.01
Chungbuk	7.881	7.731	8.034	<.01
**Main Diagnosis**				
Other main diagnosis(remaining 10 diagnoses)	1			
Diseases of the eye and adnexa (H00-H59)	1.679	1.627	1.733	<.01
Mental and behavioral disorders (F00-F99)	2.011	1.837	2.200	<.01
Diseases of the musculoskeletal system and connective tissue (M00-M99)	2.014	1.979	2.049	<.01
Diseases of the circulatory system (I00-I99)	2.096	2.064	2.129	<.01
Factors influencing health status and contact with health services (Z00-Z99)	2.925	2.879	2.972	<.01
Diseases of the nervous system (G00-G99)	2.972	2.883	3.064	<.01
Neoplasms (C00-D48)	3.987	3.936	4.038	<.01
Congenital malformations, deformations, and chromosomal abnormalities (Q00-Q99)	4.111	3.606	4.687	<.01
**Surgery**				
NOT Performed	1			
Performed	1.728	1.709	1.747	<.01
**CCI**				
0 point	1			
1–2 points	1.457	1.438	1.475	0.34
Over 3 points	2.148	2.117	2.179	<.01

Compared to Gyeongnam province (reference group), all other non-capital regions showed significantly higher odds of capital area hospitalization. Chungbuk province exhibited the highest odds (OR = 7.881), indicating its residents were nearly 7.9 times more likely to utilize capital area hospitals than those from Gyeongnam, followed by Chungnam (OR = 5.447) and Gangwon (OR = 5.389).

Several main diagnostic categories were significantly associated with increased odds of capital area hospitalization compared to ‘other main diagnoses’. Patients with congenital malformations (OR = 4.111) and neoplasms (OR = 3.987) had the highest odds of being hospitalized in the capital area. Patients who underwent surgery during hospitalization had significantly higher odds (OR = 1.728) of being hospitalized in the capital area compared to those who did not (reference group).

A higher comorbidity burden was significantly associated with increased odds of capital area hospitalization, with patients having 1–2 CCI points showing 1.457 times higher odds and those with over 3 points showing 2.148 times higher odds compared to the 0-point reference group.

## Discussion

This study investigated the patterns and associated factors of capital-area hospitalization among elderly patients residing in non-capital regions of South Korea, specifically comparing trends before (2018−2019) and during (2020−2021) the COVID-19 pandemic using weighted data from the Korea National Hospital Discharge In-Depth Injury Survey. The results revealed that the overall proportion of capital-area hospitalizations remained statistically unchanged at 7.5% in both periods although the detailed results revealed varied statistically significant shifts across demographics, geographical locations, and clinical characteristics.

The initial finding indicates that the total number of hospitalized patients aged over 65 years residing in non-capital areas experienced an 8.4% decline during the COVID-19 pandemic (2,881,091 weighted cases) compared to the pre-pandemic era. This observed decrease in overall inpatient numbers aligns with previous research reporting reduced hospital utilization among outpatients and emergency patients during the COVID-19 pandemic [14,15]. However, despite government interventions such as social distancing and regional movement restrictions implemented to mitigate the spread of COVID-19, the proportion of hospitalizations in the capital area among elderly patients from non-capital regions remained constant at 7.5% both before and during the pandemic. Consequently, the concentration of hospital utilization in the capital area persisted throughout the pandemic period.

Especially, for patients aged 65–84 years, the rate of hospitalization in the capital area either remained unchanged or even slightly increased by 0.1% following the pandemic’s onset. This sustained or increased movement to capital area hospitals by elderly patients, who are recognized as being more vulnerable to COVID-19 than other age groups, suggests a continued willingness to undertake risks for healthcare access [16]. While perceptual and structural factors—such as the perceived superiority of tertiary hospitals and the limited capacity of regional healthcare systems—were major influences, additional factors likely shaped elderly patients’ decisions during the pandemic. Medically, continuous treatment for severe or chronic diseases was often unavoidable. Behaviorally, habitual referral patterns to capital-area hospitals persisted despite mobility restrictions. Psychologically, anxiety about infection and greater trust in large tertiary hospitals over smaller local facilities further encouraged care-seeking in the capital area. Together, these factors suggest that healthcare necessity and habitual behavior outweighed infection-related concerns, sustaining capital-area hospitalization among elderly patients during COVID-19.

Regarding the main diagnosis, neoplasms (C00-D48) demonstrated an increase in capital area hospitalization by 0.5% during the COVID-19 pandemic, accounting for 27.9% of regional elderly patients during this period. This finding aligns with previous observations by Han, who reported a high concentration of hospital utilization in the capital area for gastric cancer patients between 2005 and 2009, even in the face of policy changes [23]. It appears that elderly patients from non-capital areas continued to seek cancer treatment in the capital area, despite the COVID-19 situation, particularly for diseases with high severity such as cancer.

Through logistic regression, we demonstrated that during the pandemic, factors such as female gender, younger age within the elderly cohort, residence in specific non-capital regions (e.g., Chungbuk, Chungnam), certain severe diagnoses (e.g., congenital malformations, neoplasms), the need for surgery, and a higher comorbidity burden were significantly associated with increased odds of capital-area hospitalization. Specifically, regional females aged over 65 years exhibited significantly higher odds of hospitalization in the capital area compared to males. This gender disparity aligns with previous research by Yang et al., who found that female cancer patients from non-capital areas were more likely to utilize capital area hospitals, particularly for malignant neoplasms of the female reproductive system (C51-C58) which showed the highest utilization among all neoplasms [24].

For the residential area variable, elderly patients residing in areas geographically proximate to the capital area exhibited a significantly higher frequency of hospitalization in capital-area facilities compared to those from more distant regions. The observed increase in capital-area hospitalization among Chungbuk residents may be partly explained by the province’s close proximity to the Seoul Metropolitan Area and its relatively limited tertiary healthcare infrastructure, which likely encouraged patient movement toward the capital area. By contrast, Jeonbuk, Jeonnam, and Gyeongnam provinces have lower geographical accessibility to Seoul. The decrease in hospitalization from these regions may have been influenced by both their greater distance from the capital area and the travel restrictions implemented during the COVID-19 pandemic, which particularly constrained the mobility of elderly individuals. These findings suggest that geographic accessibility and pandemic-related movement limitations jointly affected regional patterns of hospital utilization. Consistent with a previous study, the higher capital-area hospitalization rate among patients from regional areas is often driven by their self-referral to tertiary care centers, particularly among those from more affluent urban areas and in close proximity to specialized healthcare facilities [25]. That study further mentioned that the perceived inadequacy of healthcare facilities in non-capital area is a crucial factor contributing to this higher capital-area hospitalization rate among regional patients. Therefore, bolstering the capacity of regional healthcare and establishing an effective referral system are essential measures to enhance the overall efficiency of healthcare utilization.

Concurrently, regional elderly inpatients with high disease severity, exemplified by neoplasms (C00-D48), demonstrated a significantly higher frequency of hospitalization in capital area hospitals compared to those with other main diagnoses. Furthermore, the likelihood of capital-area hospitalization was significantly more frequent among non-capital area patients aged over 65 years if they underwent surgery or had a Charlson Comorbidity Index (CCI) exceeding 3 points. These findings are corroborated by Hong et al., who identified surgery and severe main diagnoses, such as neoplasms and diseases of the nervous system, as key characteristics of regional patients utilizing hospitals in the capital area [26]. Additionally, Chang noted that cancer patients often select hospitals based on factors such as the reputation of physicians and the availability of state-of-the-art medical equipment [27].

The sustained concentration of hospital utilization in the capital area continues to limit access to timely and high-quality care for residents in non-capital regions, thereby exacerbating inter-regional health disparities. However, this persistent reliance on capital-area hospitals largely stems from limited healthcare capacity in non-capital regions. Regional hospitals often face shortages of medical staff and lack advanced diagnostic equipment or tertiary-level facilities to manage complex cases, which undermines patient confidence in local services. Moreover, elderly patients tend to prefer capital-area hospitals for their reputation, perceived quality, and broader range of specialized care. Together, these factors reinforce the structural imbalance in hospital utilization between capital and non-capital regions. Addressing this structural imbalance has become a critical policy imperative to ensure equitable healthcare access across all regions. Therefore, it is essential for the government to implement innovative and regionally tailored policies aimed at strengthening the healthcare system in non-capital regions, given the persistent concentration of hospital utilization in the capital area even during the COVID-19 pandemic. These policies should prioritize targeted investments in regional healthcare infrastructure, equitable distribution of healthcare professionals, and the establishment of regional tertiary and specialized centers to decentralize advanced medical services. In parallel, hospitals in non-capital regions should adopt differentiated strategies—such as developing specialized centers for women’s and geriatric care, investing in infrastructure, and introducing advanced medical technologies—to enhance their competitiveness and better retain regional patients based on local healthcare needs. In addition, the integrated referral systems could further enhance access to specialized care for elderly patients in non-capital regions. While this study used the capital vs. non-capital regional distinction as a proxy for measuring the geographic concentration of hospital utilization, the applicability of a formally defined Hospital Service Area (HSA) framework warrants consideration in future research. In South Korea, patients retain the legal right to freely select their healthcare provider, and the existing tiered healthcare delivery system does not restrict utilization strictly within designated service area boundaries. Future studies incorporating HSA-based approaches may provide more refined catchment area definitions and improve the precision of concentration measures in the Korean healthcare context.

This study has several limitations. Firstly, while weights were applied to estimate the population, the study did not encompass a survey of all patients in Korea, which may affect the generalizability of the findings. Secondly, the focus was solely on hospitalized patients, suggesting a need to expand future research to include outpatient and emergency patients to provide a more comprehensive understanding of hospital utilization patterns. Thirdly, the findings of this study were not directly compared with those from other independent programs or regional healthcare initiatives, which may limit the external validity of our conclusions. Differences in data sources, study populations, and analytic approaches across studies made direct comparison challenging, so future research incorporating such comparative analyses would provide more stringent and comprehensive insights. Finally, continuous research is warranted to further identify the complex factors influencing the concentration of hospital utilization in the capital area, even beyond the immediate impact of the COVID-19 pandemic.

Nevertheless these limitations, this study offers significant insights into the persistent concentration of hospital utilization in the capital area among elderly patients from non-capital regions, even amidst the unprecedented public health challenges posed by the COVID-19 pandemic. This research underscores the critical need for policy interventions aimed at strengthening regional healthcare infrastructure and services to mitigate existing health disparities. Future research should expand its scope beyond hospitalized patients to encompass outpatient and emergency care, thereby providing a more comprehensive understanding of healthcare utilization patterns. Furthermore, continuous and longitudinal studies are essential to elucidate the multifaceted factors driving the concentration of hospital utilization in the capital area, even in the post-pandemic era, to inform sustainable and equitable healthcare policy development.
